# Sparse gut microbiomes in solitary bees and wasps

**DOI:** 10.1098/rsbl.2026.0459

**Published:** 2026-11-11

**Authors:** Jennifer N. Schlauch Saiyawong, Kristal M. Watrous, Stephen Buchmann, Annalie Melin, Tobin J. Hammer

**Affiliations:** ^1^ Department of Ecology and Evolutionary Biology, University of California Irvine, Irvine, CA, USA; ^2^ Ecology and Evolutionary Biology, University of Arizona, Tucson, AZ, USA; ^3^ South African National Biodiversity Institute, Cape Town, South Africa; ^4^ Nelson Mandela University, Port Elizabeth, EC, South Africa

**Keywords:** microbiota, pollinators, Hymenoptera, Masarinae, sociality, pollen-feeding, Anthophila

## Abstract

Bees and wasps are ecologically vital, but many species are declining owing to anthropogenic stressors. Social bees harbour host-specific and dense gut microbiomes that affect their resilience to stress. However, there are tens of thousands of other bee and wasp species that vary in sociality and diet (including pollen-feeding and predatory guilds), traits known to influence host–microbe symbioses. The role of gut microbes in the biology of these species is largely unknown. Here, we measured the composition and absolute abundance of bacterial communities in adult metasomas across 61 genera and 14 families of field-collected bees, predatory wasps and pollen wasps. We found that solitary bees and both wasp guilds harbour distinct bacterial taxa and lower bacterial abundances as compared with social bees. Bacterial abundances also varied extensively among and within genera of solitary bees, with little variation explained by body size, diet breadth or nesting ecology. Furthermore, microbiome composition was only weakly differentiated among solitary bees and the two wasp groups, even when comparing herbivorous (pollen-feeding) and omnivorous taxa. We suggest that the sparse and somewhat stochastic microbiomes of solitary bees and wasps reflect weak host dependence on microbially mediated functions, a trait that may influence their responses to environmental change.

## 1. Introduction

Bees and wasps are an enormously diverse group with essential roles in terrestrial ecosystems, but their persistence is threatened by anthropogenic stressors [1–4]. How these insects will fare in the future may depend, in part, on the nature of their microbiomes. Many animal taxa harbour gut microbial symbionts that improve their ability to resist diseases, pesticides, nutrient limitation or other perturbations [5–7]. Others appear to lack beneficial gut symbionts, instead harbouring only transients, commensals or pathogens [8], and may thus lack an important mechanism for adapting to changing environments [9,10]. How common these distinct symbiotic strategies are among most bees and wasps is unclear. While a few taxa have been thoroughly studied, generalizing their findings is complicated by variation in sociality and diet, traits known to shape the gut microbiome [11,12].

Much of the research on gut microbiomes of bees and wasps has focused on eusocial species, particularly honeybees (*Apis*), bumblebees (*Bombus*) and certain wasps in the family Vespidae. Adult *Apis* and *Bombus* host a highly abundant (approx. 10^8^–10^9^ cells per individual) and consistent microbial community of host-specific gut bacteria that are transmitted between generations within the colony [13–15]. This symbiosis appears to be ancient [14,15], likely reflecting host dependence on bacterially provided functions, including digestion, detoxification and pathogen defence [16]. Among wasps, one eusocial species with large and long-lived colonies, as well as an unusual diet—the honey wasp *Brachygastra mellifica*—was also found to harbour abundant and host-specific gut bacteria [17]. At least in *Apis* and *Bombus*, the best-studied of the eusocial bees and wasps, the gut microbiome is known to mediate responses to natural and anthropogenic stressors, such as pathogens or agrochemicals [18,19]. Thus, microbiome research in other bee and wasp taxa could have important implications for their conservation and management in the Anthropocene.

Over 77% of bee species and the vast majority of wasp species are solitary [1,20,21], and 16S rRNA gene sequencing data indicate that their gut microbiomes differ from those of the aforementioned eusocial species [14,22,23]. In adult solitary bees and wasps, gut bacterial communities often vary substantially in composition between conspecific individuals or populations, and their constituent taxa appear to be horizontally acquired from flowers, prey or other environmental sources [17,22,24–26]. These characteristics are consistent with a weak dependence on functions provided by bacterial symbionts [8], although counter examples exist [27]. To our knowledge, experimental data are not available to evaluate the hypothesis of weak gut-symbiotic dependence in adult solitary bees and wasps, as most species cannot be easily reared in the laboratory. Therefore, there is a need for an observational approach to broadly assess gut symbiosis in these insects.

Here we use absolute abundance—an often overlooked parameter in microbiome studies [28,29]—to infer the functional importance of gut bacterial communities to solitary bees and wasps. Absolute abundance reflects function because a low biomass of microbes in a microbiome will constrain the total metabolic or ecological outputs of that microbiome. Thus, hosts that rely upon beneficial symbionts typically provide nutrients and housing to support high levels of microbial growth and hence microbial services [30–32]. By contrast, hosts that do not invest in maintaining beneficial symbionts—those with largely transient or commensal microbes—are expected to harbour fewer microbes overall, the abundance of which may fluctuate stochastically depending on diet-derived microbial inputs or other factors [33–35]. The few studies that have quantified gut microbiomes in adult solitary bees or wasps have found low absolute abundances compared with microbiomes in eusocial species [14,17], but much broader sampling is needed to infer whether this is a general pattern.

To assess the role of sociality in shaping the gut microbiome across bees and wasps, we compared bacterial communities among field-collected solitary bees (35 genera), eusocial bees (eight genera), predatory wasps (13 solitary genera and two eusocial genera) and pollen wasps (three genera). The pollen wasps (Vespidae: Masarinae) are a small clade of ground-nesting solitary wasps and one of only two obligately pollen-feeding hymenopterans besides bees [36,37]. These were included to examine the role of diet as an additional factor shaping the microbiome. Adult-stage abdomens (technically, metasomas) were sampled, which include most of the gut (excluding the proximal oesophagus) as well as reproductive tissues, haemolymph, cuticle and other potential sites of symbiont colonization. We used 16S rRNA gene sequencing and quantitative polymerase chain reaction (qPCR) to measure bacterial community composition and absolute abundance, respectively. These are high-throughput and relatively inexpensive methods that are commonly used to characterize microbiome structure in insects [38,39]. Our work reveals large and previously unexplored variation in bacterial abundances among adult bees and wasps, at both individual- and species-level scales. Such variation has the potential to influence host fitness and the resilience of these insects in the Anthropocene.

## 2. Material and methods

### (a) Field collections

A total of 443 wild adult bee and wasp specimens were sampled for this study. Of these, 310 specimens were collected in California and Arizona from April to August of 2023. An additional 100 samples from South Africa, the Canary Islands and the western USA were included, collected between January 2022 and March 2025. These insects were caught via hand net and (except for South African specimens) immediately euthanized by collection into an individual 1.5 ml tube of 95% ethanol at room temperature. The 30 South African specimens were euthanized via a cyanide killing jar, stored at –20°C briefly, then transferred to individual 95% ethanol vials at room temperature. Thirty-three Meliponini samples, collected and stored in 95% ethanol, were included in the dataset; these samples are described in [40] and were previously sequenced and identified [41]. Following [12], specimens were not surface-sterilized prior to extraction. All non-Meliponini specimens were identified at least to genus by J.N.S.S. using keys [42,43].

### (b) Bee trait data

We measured the body size of solitary bees and a subset of eusocial bees using a 5 mm mini measuring ruler and a dissection microscope. Intertegular distance, a standard metric of body size for bees, was measured between the tegulae at the wing bases [44]. Nesting ecology of solitary bees was summarized from [1] and classified as: ground nesting, leaf-cutter, stem-nesting and brood parasite. Diet breadth of solitary bees was classified as generalist or specialist [45] (see supplemental material for more detail). Sex was determined by counting antennae or other diagnostic external features. Sample metadata are available from Dryad [46].

### (c) DNA sequencing and quantitative polymerase chain reaction

DNA was extracted from individual homogenized abdomens and PCR-amplified with V4 (515F/806R) 16S rRNA gene primers. Following established recommendations for controlling contamination [38], multiple blanks (negative controls) and mock communities (positive controls) were included. Amplicons were sequenced on four Illumina MiSeq runs (2 × 250 or 400 bp, depending on the run). Raw reads are available from NCBI (BioProject no. PRJNA1472269). Stingless bee samples from [41] were added to this dataset; these were originally extracted and PCR-amplified using the same protocol in the same laboratory, with 16S rRNA V4 amplicons sequenced on an Illumina MiSeq (2 × 250 bp). All sequence data were quality-filtered and processed with DADA2 [47] following [48]. The amplicon sequence variants (ASV) table and representative sequences are available from Dryad [46]. qPCR with V1-V2 (27F/355R) primers was used to measure absolute abundances of bacteria in the DNA extracts, following [47,49]. We multiplied the resulting total 16S copy numbers by the proportion of V4 amplicon sequences classified as non-contaminant bacteria, producing an estimate of bacterial 16S rRNA gene copies per abdomen. More details are provided in the supplemental material.

### (d) Statistical analysis

Analyses were conducted in R v. 4.3.2 [50]. To test whether chloroplast sequences, absolute abundances, the Shannon diversity index or ASV richness varied between host guilds, linear mixed-effects models were fitted with either chloroplast proportion, absolute abundance (log-transformed), Shannon diversity or ASV richness as the response, host guild as a fixed effect and host genus as a random effect. The chloroplast model used a likelihood ratio test, with proportion comparisons based on Tukey's HSD (honestly significant difference). To test whether diet, nesting ecology, body size or sex was associated with absolute abundance, identical models were fitted for solitary bees only, with the trait as the fixed effect [51,52]. To visualize microbiome composition across host guilds, we used an non-metric multidimensional scaling (NMDS) ordination of Bray–Curtis dissimilarities with vegan [53]. PERMANOVA and betadisper tests were performed to test for differences among guilds in microbiome composition and dispersion, respectively. For PERMANOVA, pairwise comparisons were conducted using pairwise Adonis [49] with false discovery rate correction. See in the supplemental material for further details.

## 3. Results and discussion

After sequence data processing, we obtained microbiome composition (16S rRNA gene sequencing) and absolute abundance (qPCR) data from 422 adult bee and wasp specimens**,** including 298 solitary bees from 35 genera in six families, 66 eusocial bees from eight genera in one family, 36 predatory wasps from 15 genera in six families and 22 pollen wasps from three genera in one family (figure 1A). This dataset likely represents the broadest (in terms of phylogenetic extent) microbiome characterization of bees and wasps investigated to date. Furthermore, the host taxa differ in sociality and diet (figure 1A), allowing an assessment of how these traits may be associated with the gut microbiome.

**Figure 1.**
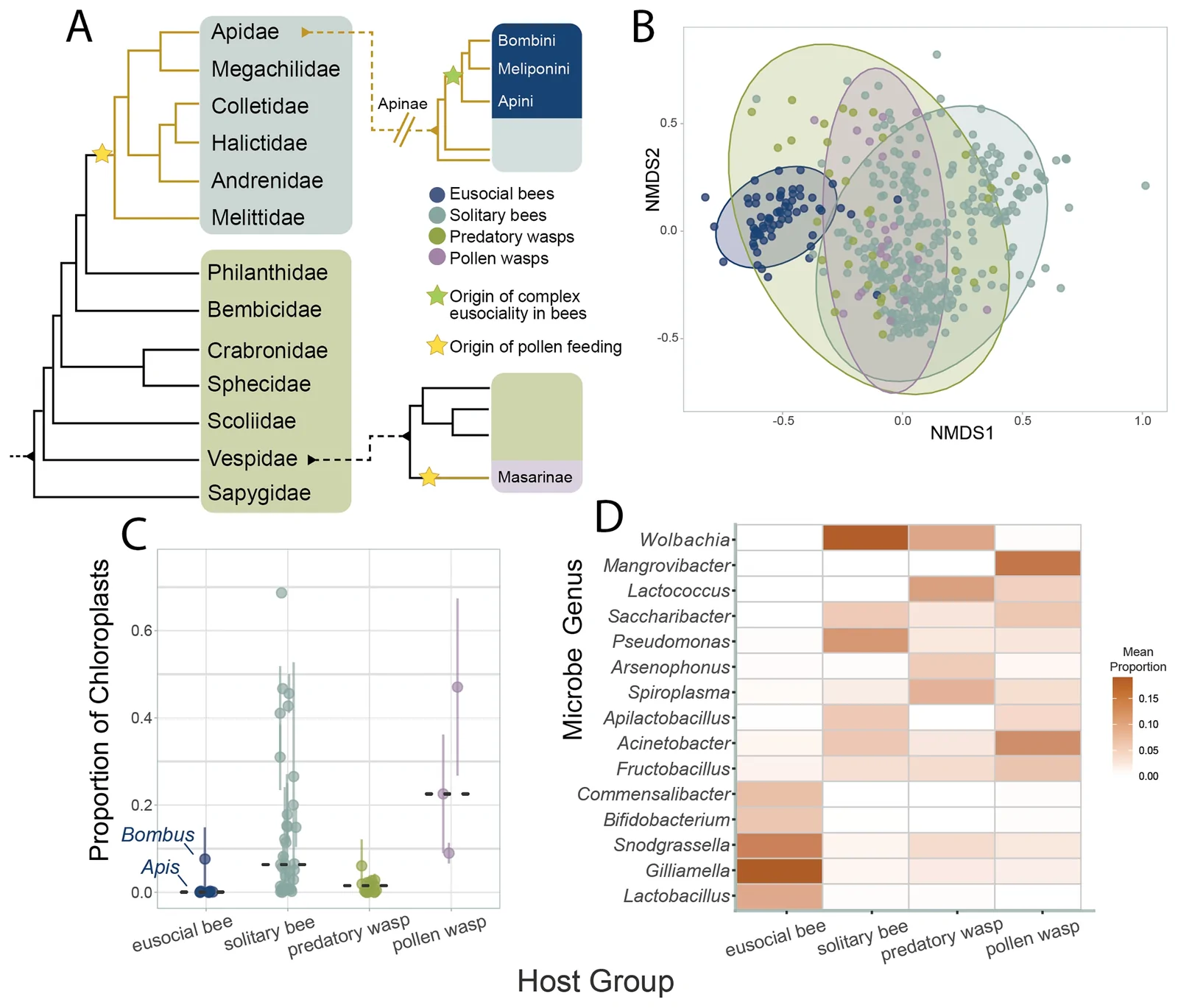
Variation in microbiome composition across adult bees and wasps. (A) Cladogram of all families included in the study, adapted from [54], indicating the origin of complex eusociality within bees and the independent origins of pollen-feeding in bees and pollen wasps. (B) NMDS ordination of Bray–Curtis dissimilarities. Each point is an individual sample (*N* = 298 solitary bees, 66 eusocial bees, 36 predatory wasps and 22 pollen wasps). Ellipses represent 95% confidence intervals around the centroid for each insect guild. (C) Chloroplast content in sequence libraries. Each point represents the mean proportion of chloroplast sequences for a given host genus; for genera with replicate individuals, the vertical line indicates the standard error of the mean. Medians for each host guild are indicated by dashed grey lines. Points are horizontally jittered. Among eusocial bees, honeybees (*Apis*) and bumblebees (*Bombus*) are labelled, but not stingless bee (Meliponini) genera (*N* = 35 solitary bee genera, eight eusocial bee genera, 15 predatory wasp genera and three pollen wasp genera). (D) Fifteen of the most common bacteria in bees and wasps, representing a combination of, for each host guild, the five bacterial genera with the highest mean proportion of sequences across samples.

Microbiome composition varied between eusocial bees, solitary bees, predatory wasps and pollen wasps (‘host guilds’) (PERMANOVA; *F* = 17.622, *R*^2^ = 0.11, *p* = 0.001; figure 1B). This result is robust to rarefaction depth: at a higher number of reads per sample (10 000) but with fewer (*N* = 316) samples, host guild was also significant (PERMANOVA *F* = 17.829, *R*^2^ = 0.15, *p* = 0.001). All host guilds differed in microbiome composition from one another (pairwise PERMANOVA; *p*-adjusted = 0.001), but eusocial bees were more different from solitary bees, and both wasp groups (in composition, *R*^2^ values all > 0.11; and dispersion, all *p* < 0.0002) than solitary bees and wasps were to each other (composition, *R*^2^ values all < 0.04; dispersion, all *p* > 0.2). By contrast, alpha diversity (Shannon diversity or ASV richness) did not significantly vary among host guilds (linear mixed effects models; Shannon $\chi^{2}$ = 0.68, *p* = 0.88; richness $\chi^{2}$ = 4.301, *p* = 0.23). The composition pattern matches previous evidence that eusocial bees have a highly distinctive gut microbiome relative to solitary bees [12,14]. By contrast, there was relatively little partitioning by diet guild, with large overlap in composition among predatory wasps and the herbivorous solitary bees and pollen wasps (figure 1B,D). In other animals, carnivores and herbivores typically have very different gut microbiome composition [11,55]; here, this may be explained by the largely herbivorous diet of predatory wasps as adults [20].

The overlap in microbiome composition between solitary bees and the two wasp guilds might be driven by horizontal transmission from common pools of environmental microbes. Most of the prevalent bacterial genera we find in solitary bees and pollen wasps (e.g. *Pseudomonas, Acinetobacter, Apilactobacillus, Saccharibacter, Fructobacillus* and *Lactococcus*; figure 1D) have been detected in other solitary bee species and environments (e.g. [23,25,56]), suggesting that our microbiome data are generally representative. These bacterial genera also frequently inhabit flowers [24,57,58]. The overlap between solitary bee-associated and flower-associated bacteria aligns with prior research demonstrating that adult solitary bees acquire gut bacteria from flowers or other environmental substrates [23,59,60]. Some of these putatively flower-derived bacteria (e.g. *Lactococcus* and *Fructobacillus*) were also common in predatory wasps (figure 1D), potentially because they often visit flowers to consume nectar or prey on florivorous arthropods [21]. *Wolbachia*, a vertically transmitted endosymbiont that frequently parasitizes insect reproductive systems [61], was also very common in solitary bees and predatory wasps (figure 1D), as expected from prior surveys of Hymenoptera [62,63]. The eusocial bee microbiomes were dominated by gut bacterial taxa generally thought to be specific to eusocial bees [14,15], especially *Snodgrassella* and *Gilliamella* (figure 1D). However, these bacteria also occurred at a low prevalence in other bee and wasp samples (figure 1D). One possibility is that they are not as restricted to eusocial bees as previously thought [64]. Another explanation is cross-contamination among samples during DNA extraction, library preparation and sequencing, an issue exacerbated by the low absolute abundance of microbes [38]. For the predatory wasps in particular, eusocial bee-specific bacteria could also be present owing to recent handling of bee prey [26].

Off-target sequences, such as chloroplasts, can provide clues into gut microbial abundance in herbivorous animals [38]. We found that the proportion of chloroplast sequences varied between host guilds (lmer; $\chi^{2}$ = 15.24, *p* = 0.002). As expected from their diet, predatory wasps have few chloroplast sequences (figure 1C). Among the remaining groups, all of which ingest pollen as adults [1,43,65], solitary bees and pollen wasps have higher proportions of chloroplast sequences than eusocial bees, although the difference was only statistically significant with pollen wasps (pairwise *post hoc* Tukey's test; *p* = 0.07 and *p* = 0.04, respectively) (figure 1C). In other herbivorous insects, high proportions of chloroplasts in sequence libraries from gut material are often associated with a low absolute abundance of bacteria [33,66].

We used qPCR to directly measure the absolute abundance of bacteria (in terms of 16S rRNA gene copies) and combined it with the sequencing data to correct for non-bacterial amplification. Total bacterial abundance varied strongly among host guilds (lmer; $\chi^{2}$ = 27.312, *p* < 0.001). Solitary bees, predatory wasps and pollen wasps harbour, on average orders of magnitude (approx. 2000 times) fewer bacteria than eusocial bees (figure 2A). This difference would likely be even larger if only gut-associated bacteria were compared. *Wolbachia*, the most common bacterium in the abdomens of solitary bees and the second-most common in predatory wasps (figure 1D), inhabits reproductive tissues rather than the gut [61]. By contrast, all dominant bacterial genera in eusocial bee abdomens (figure 1D) have been found to specifically inhabit the gut [12,14]. A large discrepancy in gut bacterial biomass between solitary bees and eusocial bees, as indicated here by qPCR, is also supported by scanning electron microscopy comparing the solitary bee *Megachile tosticauda* and honeybees [67].

**Figure 2.**
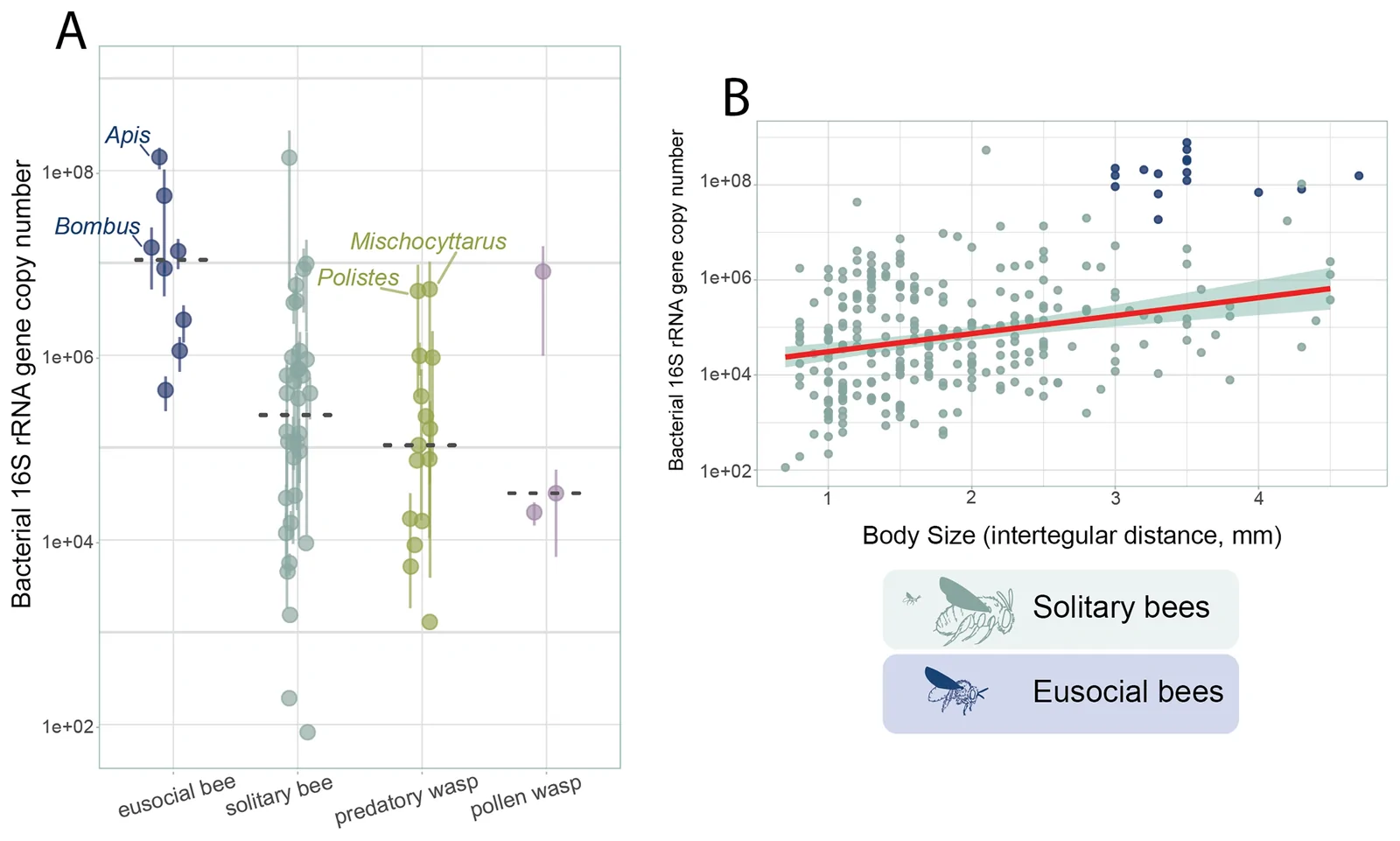
Variation in absolute abundances of bacteria across bees and wasps. (A) Bacterial 16S rRNA gene copy numbers per adult abdomen across bee and wasp guilds. The data and sample sizes are presented as in figure 1C. (B) Association between body size and bacterial 16S rRNA gene copy number for solitary bees only (*N* = 277 specimens). The red line represents a linear regression fit to the solitary bee data. A subset of eusocial bee specimens (*N* = 15 *Apis*, 1 *Bombus*) is included for reference to illustrate their body size to copy number relationship, but these data are not included in the regression model.

Solitary bees, predatory wasps and pollen wasps also exhibit orders-of-magnitude levels of variability in bacterial abundance, both within and among genera (figure 2A). We examined whether such variability could be explained by host traits, focusing on solitary bees, for which we had the largest sample size and the most trait information. At the level of individual specimens, solitary bee body size is positively, but only weakly, associated with bacterial abundance (lmer; $\chi^{2}$ = 17.46, *p* < 0.001, *R*^2^ = 0.080; figure 2B). At the level of genera, neither diet breadth (lmer; $\chi^{2}$ = 2.939, *p* = 0.230) nor nesting ecology (lmer; $\chi^{2}$ = 2.5645, *p* = 0.5573) is associated with bacterial abundance. One possible driver of this apparently stochastic variation is that individuals with especially high bacterial abundances were infected by opportunistic pathogens or parasites. Alternatively, if most of the microbiome is acquired from the environment, as suggested above, and its growth is not tightly regulated by the host, then an individual bee or wasp's microbial load may simply reflect the microbial load of its most recent meal [33]. We did find that, for a subset of seven solitary bee genera with ≥5 female and ≥5 male samples, bacterial abundances are overall slightly higher in females than in males (lmer; $\chi^{2}$ = 17.319, *p* < 0.001; supplementary material, figure S2). Female and male solitary bees may differ in morphology, feeding behaviour and other traits [1], which may explain the difference in bacterial load.

We argue that the low and variable total bacterial abundances observed in solitary bees, predatory wasps and pollen wasps likely reflect a generally weaker dependence on gut symbionts as compared with eusocial bees. Why might eusociality affect symbiotic dependence? One possibility is that the challenges of living in large groups, such as greater disease risk or nutritional demands, increase the selective pressure to maintain beneficial symbionts that help hosts meet those challenges [41]. Overlap of adult generations, a defining characteristic of eusociality [68], also provides a ready-made route for intergenerational transmission of microbes, in turn enabling the kind of persistent, reciprocal interaction that facilitates the evolution of obligate host–microbe mutualism [14,69].

As only two predatory wasp genera in our sample set, *Polistes* and *Mischocyttarus*, are eusocial (with the rest being solitary), we cannot conclusively test the effect of sociality on gut microbiome abundance within wasps. However, we note that the absolute abundances of bacteria in these two genera were higher than those of all other predatory wasp taxa (figure 2A). A previous study found that eusocial, predatory *Polistes* wasps had gut bacterial abundances slightly higher than solitary wasps, but still much lower than those of eusocial honey wasps [17]. Among ants, all of which are eusocial, many omnivorous species have low-abundance gut microbiomes [29,70]. At least among Hymenoptera, perhaps the evolution of both specialized diets and eusociality are necessary to promote host dependence on (and investment in) beneficial gut bacterial symbionts.

It may be surprising that herbivorous insects like solitary bees and pollen wasps could exist without large numbers of symbionts, given that herbivory often requires microbes to supply digestive enzymes or missing nutrients [71]. However, endogenous (host-encoded) biochemical mechanisms for pollen digestion have been described in bees [72–74], and a nectar-pollen diet is considered to be a nutritionally complete resource for insects [75]. Indeed, sterile honeybees and bumblebees have been shown to exhibit survival rates similar to those harbouring their core gut bacterial symbionts [19,76], whereas a lack of pollen nutrients does decrease adult survival [77]. This indicates that a gut microbiome is not strictly required for pollen digestion or adequate nutrition.

A weak dependence on a gut microbiome, whether for pollen-feeding or other functions, could influence the resilience of solitary bees and wasps in a changing environment. On the one hand, a lack of abundant, beneficial gut symbionts may mean that these insects are missing a source of plasticity that could help them deal with changing environments [78,79]. For example, eusocial bee gut microbiomes provide pathogen resistance [5,18], and pathogens are a major contributor to bee declines [80]. On the other hand, solitary bees and wasps may be less affected by certain stressors than strongly microbiome-dependent insects. Microbial symbionts can have physiologies that are uniquely sensitive to particular stressors, acting as an ‘Achilles’ heel’ for their host [81]. For example, heat-sensitive gut symbionts constrain the thermal tolerance of stinkbugs [82], and some of the core honeybee gut symbionts are inhibited by the herbicide glyphosate [19]. Manipulative experiments will likely be needed to determine which of these scenarios applies to solitary bees and wasps and with which kinds of stressors.

There are important caveats to our findings. Our analyses were limited to adults, because immature stages are much more difficult to sample from a broad range of species. In some bee taxa (including honeybees), developing larvae appear to have a low-abundance and/or diet-derived microbiome [12,25,83,84]. Thus, in some solitary bees and wasps, it is possible that neither larvae nor adults strongly depend on gut symbionts. However, this does not necessarily apply to all species or life stages [85,86]. Further, bees and wasps are phylogenetically and ecologically diverse, and our sample set represents only a small fraction of this biodiversity. Finally, our quantitative data are limited to the number of bacterial 16S rRNA copies, but cell numbers, live biomass, metabolic activity and other parameters should be directly measured in future work. Nevertheless, our findings provide a novel null hypothesis for future research on solitary bees and wasps: that these insects generally harbour sparse gut microbiomes that provide minimal benefits to their host.

## Supplementary Material



## Data Availability

Raw sequence data, metadata and code files are available from NCBI (BioProject no. PRJNA1472269) and Dryad [46]. Supplementary material is available online [87].
