# Supplementary material for: Sparse gut microbiomes in solitary bees and wasps
Source: Biol Lett. 2026 Nov 11;22(11):20260459. doi: 10.1098/rsbl.2026.0459 (PMC13752745; doi:10.1098/rsbl.2026.0459)
Supplement: Supplementary file 1 [file rsbl.2026.0459.zip › rsbl.2026.0459/rsbl20260459_si_001.docx]

**Supplemental Information for: Sparse gut microbiomes in solitary bees and wasps**

Jen N. Schlauch Saiyawong^1^, Kristal M. Watrous^1^, Stephen L. Buchmann^2^, Annalie Melin^3, 4^, Tobin J. Hammer^1^

**Supplemental Methods**

*Diet Breadth Data*

We analyzed the relationship between diet breadth and microbiome abundance for solitary bees collected in California and Arizona. Diet breadth for solitary bees cannot always be confidently determined at the genus level because congeneric species sometimes vary in diet breadth. Some bees in our sample set were identified to the species level, but others to the genus level only. To classify bees into diet breadth categories, we used the list of the Specialist Bees of the Western United States, compiled by Jarrod Fowler [1]. We used the definition of "specialist" according to Fowler: foraging on one plant family or on one or a few related plant genera. If a bee genus from our sample set was included in Fowler’s list of specialists, it was labelled as a "specialist," with the exceptions of *Ceratina*, *Hylaeus*, and *Lasioglossum*, of which we had several species known to not be the specialist(s) included in Fowler’s list. Final categorizations are shown in Supplemental Table 1.

*DNA Extraction and Sequencing*

In order to identify specimens to the lowest taxonomic level possible, whole insects were identified one at a time via dissection scope. Specimens were kept wet with 95% ethanol to prevent desiccation and DNA degradation. After taxonomic identification, metasomas were removed and placed in individual tubes containing 800µL ZymoBIOMICS lysis solution (Zymo Research), homogenized with a pestle, then added to ~0.5mL autoclaved zirconia silica beads (BioSpec) and two 5mm 440C stainless steel grinding balls. Samples were homogenized for six minutes with 1 minute rests every 2 minutes using a Benchmark BeadBlaster 96 homogenizer. Large pieces of integument were removed with sterile forceps, and integument pieces were avoided when pipetting out sample material for DNA extraction. Sample homogenates were then stored at -70ºC until extraction of genomic DNA (gDNA) with the ZymoBIOMICS DNA 96 kit. Following best practices for controlling contamination during gDNA extraction and library preparation [[1]](https://www.zotero.org/google-docs/?9cDu74), 28 negative controls (blanks) and 13 replicates of the ZymoBIOMICS Microbial Community Standard (Zymo Research) were included and sequenced alongside the samples. gDNA was PCR-amplified using barcoded universal primers (515F/806R) targeting the V4 region of the 16S rRNA gene, following the V.1 protocol used by the Earth Microbiome Project [[2]](https://www.zotero.org/google-docs/?XIQZEm). Clean-up and normalization were performed using the SequalPrep kit (Applied Biosystems). Pooled amplicons were run on four separate sequencing runs: two Illumina MiSeq (2 x 400 bp paired end reads) runs at the University of California Irvine Genomics Research and Technology Hub (UCI GRTH), one Illumina MiSeq (2 x 250 bp paired end reads) at UCI GRTH, and one Illumina MiSeq (2 x 250 bp) run at the University of Colorado Boulder CIRES Microbial Community Sequencing Lab. Raw reads are available on NCBI (BioProject #PRJNA1472269)

*Sequence data processing*

Raw sequences were processed using a previously described protocol [[3]](https://www.zotero.org/google-docs/?UpiETm). Briefly, DADA2 [[4]](https://www.zotero.org/google-docs/?dcxZod) was used to trim and quality-filter reads (maxEE = 2, truncQ = 2, truncLen = 240 fwd and 200 rev), infer amplicon sequence variants (ASVs), and remove chimeric ASVs. The RDP Naive Bayesian Classifier [[5]](https://www.zotero.org/google-docs/?UFNjCj) was used to assign taxonomy to the ASV table, with the SILVA 138.2 SSU rRNA reference database [[6]](https://www.zotero.org/google-docs/?cHQUAd). The ASV table and representative sequences are available through Dryad [[7]](https://www.zotero.org/google-docs/?LKB2qr) (DOI: 10.5061/dryad.4qrfj6qt2).

The ASV table was loaded into R version 4.3.2 and further analyzed using the mctoolsr R package (https://github.com/leffj/mctoolsr). Samples with zero reads were dropped, leaving 481 samples. We filtered out ASVs with fewer than 10 reads across all samples (5,587 removed), ASVs without a classification to the domain level (18 removed), as well as mitochondria, chloroplasts, and other eukaryotic ASVs (1,114 ASVs removed). We used data from the mock communities to validate the accuracy of our sequencing. The top ten ASVs, in order of mean relative abundance across the 13 samples, were: *Escherichia-Shigella, Bacillus, Salmonella, Pseudomonas, Limosilactobacillus, Listeria, Salmonella, Enterococcus, Staphylococcus*, and *Candidatus Schmidhempelia*. All of these taxa are in the mock community except for *Schmidhempelia*, a bumblebee-specialized bacterium, which probably occurred in the mock communities due to cross-contamination from bumblebee samples during DNA extraction. Contaminant ASVs were identified using the isContaminant function in the decontam package [[8]](https://www.zotero.org/google-docs/?f1hAS9) with the "prevalence" method, resulting in 91 out of 8992 ASVs classified as contaminants, which were then removed from the dataset. We then curated the taxonomy of the ASVs to avoid lumping different taxa with "NA" as genus by merging their "unid" names with the order and family names. The mean library size of abdomen samples was 23,356; to maximize the number of samples available for analysis, samples were rarefied (randomly subsampled) to 500 sequences each, resulting in 422 samples (12 removed, see Supp Fig. 1). Because the libraries originated from multiple sequencing runs with different read lengths, we summarized the ASV table to the genus level for further analyses.

*Absolute abundance*

Quantitative PCR (qPCR) was performed to measure 16S rRNA gene copy numbers. We used universal 27F/355R primers targeting the V1-V2 region of the 16S rRNA gene, following a previously described protocol [[9]](https://www.zotero.org/google-docs/?7OF5D3), and a qTOWER3 system (Analytik Jena). Reactions were run in 10µL volumes in triplicate, using PowerUp SYBR Green Master Mix (ThermoFisher) and 1µL of template DNA per reaction, with a lower limit of quantification, based on the standard curve, of 100 16S rRNA gene copies per gDNA sample. Nine negative control samples (two PCR no-template controls and seven extraction blanks) were included; the mean copy number was subtracted from the copy numbers from the insect abdomen samples. To calculate total 16S copy numbers per abdomen, the mean copy number per uL across triplicates was multiplied by 20 (as gDNA was eluted in 20uL) and by 4 (as only ¼ of each abdomen homogenate was used in the extraction). Finally, for each sample, the latter value was multiplied by the proportion of amplicon sequences classified as non-contaminant bacteria, producing an estimate of the number of bacterial 16S rRNA gene copies per abdomen. Some error may be introduced by using the V4-region sequencing data to correct the V1/V2-region 16S rRNA gene copy number data. However, our results are robust to whether or not this correction is made. Raw and corrected copy numbers are strongly correlated across all samples (R^2^ > 0.968 when comparing log-transformed values).

*Statistical Analyses*

Statistical analyses were conducted in R v. 4.3.2 (R Core Team, 2023). Linear mixed-effects models were fitted using the lmer() function in lme4 (version 2.0.1) [[10]](https://www.zotero.org/google-docs/?FENFxv). We tested the significance of fixed effects with Wald χ² tests with type III sums of squares (car::Anova version 3.1.5 [[11]](https://www.zotero.org/google-docs/?dXFMIr)), except for the chloroplast models, which used likelihood ratio tests (lme4::anova, Bates et al. 2015). Comparisons of chloroplast proportions were based on pairwise post hoc Tukey’s HSD tests, using the effect sizes of the estimated marginal means of host guilds (emmeans::pairs, version 2.0.4 [[3]](https://www.zotero.org/google-docs/?QKeUii)). To test whether chloroplast sequences or absolute abundances varied between host guilds, separate linear mixed-effects models were fitted with either chloroplast proportion or absolute abundance (log-transformed) as the response, host guild as a fixed effect, and host genus as a random effect. To calculate alpha diversity for samples, we used mctoolsr::calc_diversity (<https://github.com/leffj/mctoolsr>; [[12]](https://www.zotero.org/google-docs/?up9P9X)) to calculate the Shannon diversity index and ASV richness to test whether these differed between host guilds. Separate linear mixed-effects models were fitted with either Shannon diversity or ASV richness as the response, host guild as a fixed effect, and host genus as a random effect to test whether diversity or richness each differed significantly among guild.

To test whether diet or nesting ecology was associated with absolute abundance, identical linear mixed-effects models were fitted for solitary bees only, with diet or nesting ecology as the singular fixed effect and host genus as a random effect. To test the effect of bee body size (ITD) on absolute abundance, a linear model was generated for solitary bees only with absolute abundance (log-transformed) as the response and ITD as the predictor. To test whether sex of the individual was associated with absolute abundance for solitary bees, we analyzed a subset of solitary bee genera for which sex had been determined and specimens included at least five of each sex.

To visualize microbiome composition across host guilds, we used non-metric multidimensional scaling ordination (NMDS) of Bray–Curtis dissimilarities calculated with the vegan package (version 2.7.5) [[13]](https://www.zotero.org/google-docs/?aZ75rh). A permutational analysis of variance (PERMANOVA) with 999 permutations was performed to test for differences among guilds in composition. Pairwise comparisons were conducted using pairwiseAdonis [[14]](https://www.zotero.org/google-docs/?lgvrwr) with False Discovery Rate correction. To test dispersion differences, we used the betadisper test from the vegan package, then pairwise post hoc Tukey's HSD tests on the results. All package versions and code can be found in the RMarkdown file of the Dryad dataset: <https://doi.org/10.5061/dryad.4qrfj6qt2>.

| **Genus** | **Total Specimens in Main Analysis** | **Diet Breadth** | **Specimens used in Trait Analysis** |
| --- | --- | --- | --- |
| **Solitary Bees** | | | |
| *Agapostemon* | 4 | generalist | 4 |
| *Andrena* | 2 | specialist | 2 |
| *Anthophora* | 15 | generalist | 11 |
| *Anthophorula* | 1 | specialist | 1 |
| *Ashmeadiella* | 4 | specialist | 4 |
| *Brachymelecta* | 1 | brood parasite | 1 |
| *Calliopsis* | 16 | specialist | 16 |
| *Centris* | 13 | specialist | 8 |
| *Ceratina* | 20 | generalist | 20 |
| *Coelioxys* | 1 | brood parasite | 1 |
| *Diadasia* | 6 | specialist | 1 |
| *Dufourea* | 12 | specialist | 12 |
| *Epeolus* | 6 | brood parasite | 6 |
| *Epimelissodes* | 6 | specialist | 6 |
| *Ericrosis* | 2 | brood parasite | 2 |
| *Eucera* | 2 | specialist | 2 |
| *Exomalopsis* | 21 | specialist | 21 |
| *Halictus* | 4 | generalist | 4 |
| *Hesperapis* | 3 | specialist | 3 |
| *Hoplitis* | 1 | specialist | 1 |
| *Hylaeus* | 19 | generalist | 16 |
| *Lasioglossum* | 64 | generalist | 64 |
| *Megachile* | 10 | specialist | 10 |
| *Megandrena* | 1 | specialist | 1 |
| *Melissodes* | 18 | specialist | 18 |
| *Nomada* | 2 | brood parasite | 2 |
| *Oreopasites* | 11 | brood parasite | 11 |
| *Paranthidium* | 3 | specialist | 3 |
| *Perdita* | 16 | specialist | 16 |
| *Protandrena* | 1 | specialist | 1 |
| *Sphecodes* | 1 | brood parasite | 1 |
| *Trachusa* | 4 | specialist | 4 |
| *Triepeolus* | 5 | brood parasite | 5 |
| *Xylocopa* | 2 | generalist | 2 |
| *Zacosmia* | 1 | brood parasite | 1 |
| **Eusocial Bees** | | | |
| *Apis* | 26 | generalist | 15 |
| *Bombus* | 7 | generalist | 1 |
| *Lestrimelitta* | 5 |  |  |
| *Michmelia* | 5 |  |  |
| *Oxytrigona* | 5 |  |  |
| *Tetragona* | 5 |  |  |
| *Tetragonula* | 8 |  |  |
| *Trigona* | 5 |  |  |
| **Omnivorous wasps** | | | |
| *Ammophila* | 2 |  |  |
| *Bembix* | 6 |  |  |
| *Campsomeris* | 2 |  |  |
| *Cerceris* | 2 |  |  |
| *Chalybion* | 1 |  |  |
| *Delta* | 1 |  |  |
| *Dielis* | 1 |  |  |
| *Eucerceris* | 1 |  |  |
| *Eumenes* | 1 |  |  |
| *Mischocyttarus* | 5 |  |  |
| *Pepsis* | 1 |  |  |
| *Philanthus* | 4 |  |  |
| *Polistes* | 4 |  |  |
| *Sceliphron* | 1 |  |  |
| *Triscolia* | 4 |  |  |
| **Pollen Wasps** | | | |
| *Ceramius* | 18 |  |  |
| *Masarina* | 2 |  |  |
| *Pseudomasaris* | 2 |  |  |

Supplemental Table 1: The genera included in the analysis, grouped by host guild, with their sample sizes. For the trait analyses (diet breadth and body size), only solitary bees were used, with a subset of *Apis* and *Bombus* included as references for eusocial bees.

| **Solitary Bees** | |
| --- | --- |
| Western United States (California, Arizona) | 292 |
| Canary Islands (Spain) | 6 |
| **Eusocial Bees** | |
| Australia | 8 |
| Brazil | 14 |
| Canary Islands (Spain) | 12 |
| Colombia | 2 |
| Costa Rica | 3 |
| Peru | 6 |
| Western United States (California, Arizona) | 21 |
| **Omnivorous Wasps** |  |
| South Africa | 9 |
| Western United States (California, Arizona) | 27 |
| **Pollen Wasps** |  |
| South Africa | 20 |
| Western United States (California, Arizona) | 2 |

Supplemental Table 2: The regions from which the host guilds were collected, with the final analyzed sample sizes (number of individuals).

Supplemental Figure 1: Rarefaction curve for samples. Graph made using filtered, cleaned, but unrarefied data from 434 samples. After setting a rarefaction depth of 500, 422 samples remained. Due to overplotting, many more samples are in the lower portion of the graph but are obscured.

Supplemental Figure 2: Total bacterial 16S rRNA gene copy numbers (log scale) between female and male solitary bees. These data only include solitary bee genera whose sex was determined (via visual identification during species identification) and there were five or more individuals for each sex. Dashed lines represent the median copy number in individuals of that sex.

[1.](https://www.zotero.org/google-docs/?vFMRsV)	[Williamson EM, Hammer TJ, Hogendoorn K, Eisenhofer R. 2025 Blanking on blanks: few insect microbiota studies control for contaminants. *mBio* **16**, e02658-24. (doi:10.1128/mbio.02658-24)](https://www.zotero.org/google-docs/?vFMRsV)

[2.](https://www.zotero.org/google-docs/?vFMRsV)	[Caporaso JG *et al.* 2023 EMP 16S Illumina Amplicon Protocol V.2. (doi:https://dx.doi.org/10.17504/protocols.io.kqdg3dzzl25z/v2)](https://www.zotero.org/google-docs/?vFMRsV)

[3.](https://www.zotero.org/google-docs/?vFMRsV)	[Lenth RV *et al.* 2026 emmeans: Estimated Marginal Means, aka Least-Squares Means.](https://www.zotero.org/google-docs/?vFMRsV)

[4.](https://www.zotero.org/google-docs/?vFMRsV)	[Callahan BJ, McMurdie PJ, Rosen MJ, Han AW, Johnson AJA, Holmes SP. 2016 DADA2: High-resolution sample inference from Illumina amplicon data. *Nat. Methods* **13**, 581–583. (doi:10.1038/nmeth.3869)](https://www.zotero.org/google-docs/?vFMRsV)

[5.](https://www.zotero.org/google-docs/?vFMRsV)	[Wang Q, Garrity GM, Tiedje JM, Cole JR. 2007 Naïve Bayesian Classifier for Rapid Assignment of rRNA Sequences into the New Bacterial Taxonomy. *Appl. Environ. Microbiol.* **73**, 5261–5267. (doi:10.1128/AEM.00062-07)](https://www.zotero.org/google-docs/?vFMRsV)

[6.](https://www.zotero.org/google-docs/?vFMRsV)	[Quast C, Pruesse E, Yilmaz P, Gerken J, Schweer T, Yarza P, Peplies J, Glöckner FO. 2013 The SILVA ribosomal RNA gene database project: improved data processing and web-based tools. *Nucleic Acids Res.* **41**, D590–D596. (doi:10.1093/nar/gks1219)](https://www.zotero.org/google-docs/?vFMRsV)

[7.](https://www.zotero.org/google-docs/?vFMRsV)	[Schlauch Saiyawong J, Hammer T, Watrous K, Buchmann S, Melin A. 2026 Data from: Sparse gut microbiomes in solitary bees and wasps. , 25916221 bytes. (doi:10.5061/DRYAD.4QRFJ6QT2)](https://www.zotero.org/google-docs/?vFMRsV)

[8.](https://www.zotero.org/google-docs/?vFMRsV)	[Davis NM, Proctor DM, Holmes SP, Relman DA, Callahan BJ. 2018 Simple statistical identification and removal of contaminant sequences in marker-gene and metagenomics data. *Microbiome* **6**, 226. (doi:10.1186/s40168-018-0605-2)](https://www.zotero.org/google-docs/?vFMRsV)

[9.](https://www.zotero.org/google-docs/?vFMRsV)	[Hammer TJ, Easton-Calabria A, Moran NA. 2023 Microbiome assembly and maintenance across the lifespan of bumble bee workers. *Mol. Ecol.* **32**, 724–740. (doi:10.1111/mec.16769)](https://www.zotero.org/google-docs/?vFMRsV)

[10.](https://www.zotero.org/google-docs/?vFMRsV)	[Bates D, Mächler M, Bolker B, Walker S. 2015 Fitting Linear Mixed-Effects Models Using **lme4**. *J. Stat. Softw.* **67**. (doi:10.18637/jss.v067.i01)](https://www.zotero.org/google-docs/?vFMRsV)

[11.](https://www.zotero.org/google-docs/?vFMRsV)	[Fox J, Weisberg S. 2026 *An R Companion to Applied Regression*. Third. Thousand Oaks, CA: Sage. See https://www.john-fox.ca/Companion/.](https://www.zotero.org/google-docs/?vFMRsV)

[12.](https://www.zotero.org/google-docs/?vFMRsV)	[Leff JW. 2022 mctoolsr: Microbial community analysis tools in R.](https://www.zotero.org/google-docs/?vFMRsV)

[13.](https://www.zotero.org/google-docs/?vFMRsV)	[Oksanen J *et al.* 2026 vegan: Community Ecology Package.](https://www.zotero.org/google-docs/?vFMRsV)

[14.](https://www.zotero.org/google-docs/?vFMRsV)	[Arbizu PM. 2026 pmartinezarbizu/pairwiseAdonis.](https://www.zotero.org/google-docs/?vFMRsV)
